# A Fortunate Man

**Published:** 1968-04

**Authors:** Frank Bodman


### A FORTUNATE MAN

BERGER

(Alan Lane 1967)

This illustrated essay should be of interest to members of the Society as the Fortunate Man trained at the Bristol School some twenty years ago : Under the nom de plume of Sassall the author describes in detail the life of a single handed practitioner in the Forest of Dean and philosophises on the doctor-patient relationship in a situation far removed from current practice in urban conditions.

That it is still possible to follow in the footsteps of Schweitzer without going to Africa may perhaps surprise some of us though Sassall's surgery is probably better equipped than the hospital at Lambarene.

Berger analyses the dialectical process between doctor and patient and sees it as both a process of projection and introjection on the part of the doctor who identifies so closely with his patients. The evolution of this personal interchange from the heroic intervention at crises to the more mature long term relationship is well brought out. One is reminded of Professor Halmos 'Faith of the Counsellors'—where he demonstrates that social workers have in the choice of their profession abandoned the programmes of politics and in spite of a theoretical training in scientific objectivity find themselves practising a controlled relationship with their clients, which while avoiding over identification is therapeutic because of the mutual trust and confidence established.

That this process is not without cost to the practitioner is clear from the author's account. And Sassall's periods of depression indicate the difficulties of over identification and the inevitable change and development of the personality involved in such relationships.

This essay is provocative of thought. The illustrations of places and people powerfully reinforce the problems stated.

FRANK BODMAN.

